# Unmasking MEGF10 Myopathy: A Rare Cause of Sudden Respiratory Failure in a Young Adult

**DOI:** 10.1155/crnm/6904563

**Published:** 2025-11-11

**Authors:** Benedict Kleiser, Luise Hackenbruch, Jens Schittenhelm, Antje Bornemann, Tobias Haack, Alexander Grimm, Pascal Martin

**Affiliations:** ^1^Department of Epileptology, Hertie-Institute for Clinical Brain Research, University of Tübingen, Tübingen, Germany; ^2^Department of Neural Dynamics and Magnetoencephalography, Hertie-Institute for Clinical Brain Research, University of Tübingen, Tübingen, Germany; ^3^MEG-Center, University of Tübingen, Tübingen, Germany; ^4^Institute of Medical Genetics and Applied Genomics, University of Tübingen, Tübingen, Germany; ^5^Department of Neuropathology, Institute of Pathology and Neuropathology, University Hospital of Tuebingen, Universitiy of Tübingen, Tübingen, Germany; ^6^Center for Rare Diseases, University of Tübingen, Tübingen, Germany; ^7^Genomics for Health in Africa (GHA), Africa-Europe Cluster of Research Excellence (CoRE), Tübingen, Germany

## Abstract

Sudden respiratory insufficiency is commonly attributed to cardiopulmonary causes but may also herald underlying neuromuscular disorders. In this context, rare diseases in particular pose significant diagnostic challenges. Here, we report on a 27-year-old woman who presented with unexplained respiratory insufficiency, proximal muscle pain, and weakness. Initially, she was found unconscious with severe hypoxemia (oxygen saturation 41%), low respiratory rate (4/min), and hypotension, requiring emergency intubation. After treatment for pneumonia, persistent hypercapnia and hypoxemia were noted. Two months later, she reported muscle pain, reduced strength when climbing stairs, and swallowing difficulties. Physical examination showed symmetrical proximal paresis in arms and legs (MRC 4/5), dependence on hand support for head lifting, and inability to rise from a squat unaided. Reflexes were symmetrically reduced. There were no signs of myotonia. Medical history included kyphoscoliosis; family history was noncontributory for muscular disorders. In this case, we provide guidance on navigating the multiplicity of neuromuscular differential diagnoses in case of respiratory failure in combination with peripheral weakness, leading to the final diagnose of MEGF10 myopathy in this case.

## 1. Introduction

Sudden onset of respiratory insufficiency may result not only from internal medicine conditions such as pulmonary embolism or exacerbation of pre-existing pulmonary diseases but also from a wide range of neuromuscular disorders, including myasthenia, motor neuron diseases, and myopathies [1]. Genetically determined myopathies, when considered collectively, are not uncommon; however, the individual disease entities are rare to very rare, rendering their precise diagnosis classification challenging, as in the case presented for MEGF10 myopathy. MEGF10-related myopathy is an ultrarare autosomal-recessive disorder. Reliable epidemiological data on its incidence and prevalence are currently lacking, reflecting the very limited number of cases reported to date. The condition presents with variable onset from childhood to adulthood, with clinical features including muscle weakness, dysphagia, cleft palate, and scoliosis [2–4]. Dyspnea and respiratory failure may also occur during the disease course [2, 4], although these are not always part of the initial symptoms leading to medical evaluation (e.g., [3]). In the following, we present the patient journey of a 27-year-old girl with MEGF10 myopathy that seems to manifest without evident prodromal symptoms with sudden respiratory failure.

## 2. Case

A 27-year-old female was referred for neurological evaluation due to unexplained respiratory insufficiency, proximal muscle pain, and weakness. She was initially found unconscious with cyanosis, a respiratory rate of 4/min, systolic blood pressure of 40 mmHg, and oxygen saturation of 41%, requiring emergency intubation. The initial evaluation during the multidisciplinary intensive care unit (ICU) stay revealed no explanatory internal medicine cause such as pulmonary embolism or exacerbation of pre-existing pulmonary diseases. Transesophageal echocardiography initially suggested an indeterminate mass in the right atrium, without evidence of relevant valvular abnormalities. Subsequent cardiac MRI revealed a prominent coronary sulcus between the right atrium and right ventricle, explaining the previously observed indeterminate finding and showing no evidence of thrombus. After treatment for subsequently developed pneumonia, recurrent asymptomatic hypoxemia (pO_2_ as low as 33 mmHg) and persistent hypercapnia were noted. Therefore, the patient was initially further managed by our pulmonology colleagues. Initial body plethysmography demonstrated a total lung capacity (TLC) of 2.5 L (predicted 4.8 L), a forced vital capacity (FVC) of 0.5 L (predicted 3.7 L), and a residual volume (RV) of 2.0 L (predicted 1.33 L). Respiratory manometry revealed an occlusion pressure 0.1 s (P0.1) of 0.14 kPa (normal > 0.13 kPa) but a markedly reduced maximal inspiratory pressure (MIP) of up to 1.55 kPa (normal > 7.0 kPa), suggesting possible inspiratory muscle weakness. However, central hypoventilation syndrome was also considered, but both cranial MRI and sequencing of *PHOX2B* yielded normal results. A pre-existing scoliosis was not considered a plausible competing cause, and no evidence of sleep apnea was detected during intensive care monitoring or in the setting of a lean body habitus. Overall, the respiratory symptoms could not be definitively explained at this stage.

Two months later, she reported muscle pain in arms and legs, reduced strength when climbing stairs, and occasional swallowing difficulties. Therefore, she was scheduled for outpatient neurological evaluation. The examination revealed symmetrical, proximally pronounced paresis with the muscle strength of 4/5 according to the MRC scale in arms and legs, as well as during head rotation on both sides. When lifting the head from the examination table, the patient had to use her hand for support. She was unable to stand up from a deep squat without using her hands for assistance. Reflexes were symmetrically reduced, and there were no signs of myotonia. Her medical history included kyphoscoliosis with mild thoracic asymmetry. Family history was unremarkable regarding muscular disorders.

**Figure 1:**
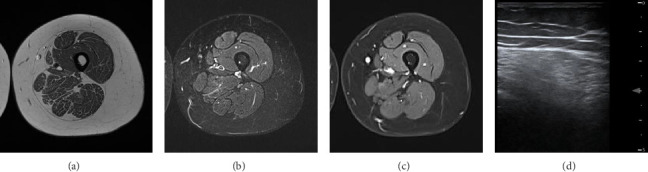
MRI of the thigh muscles in the transverse plane in (a) T1, (b) T2-TRIM and (c) Dixon with contrast agent. (d) Transversal sonography of the M. rectus femoris, vastus lateralis, right, revealing hyperechogenicity (modified Heckmatt Scale [5]: 2/4).

**Figure 2:**
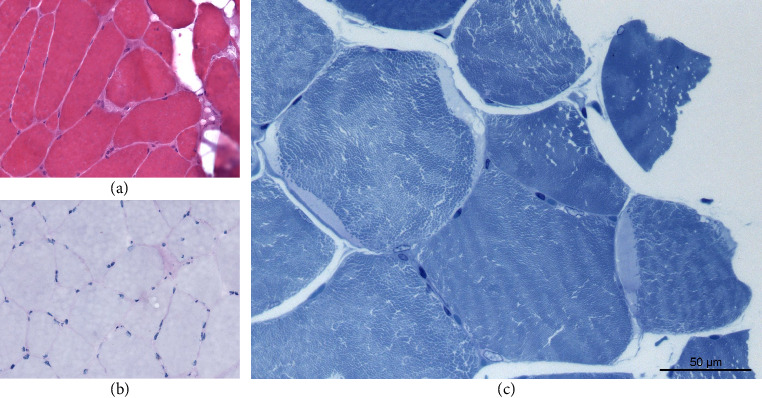
Skeletal muscle biopsy. (a) Nonspecific neurogenic variations in muscle fiber caliber (hematoxylin and eosin stain × 200). (b) Negative acid phosphatase stain, × 200. (c) Marginal, crescent-shaped, pale blue-gray homogeneous deposits (toluidine blue).

Laboratory findings revealed elevated creatine kinase levels at 876 U/L (maximum 1248 U/L; normal < 170 U/L), indicative of muscle cell breakdown. Elevated creatine kinase levels are not necessarily indicative of a primary muscle disorder and can occur in dimensions of several hundred U/L in other conditions like peripheral nerve disorders as Guillain–Barré syndrome (GBS) or critical illness polyneuropathy (CIP). Nerve conduction studies were conducted, which show normal amplitudes and conduction velocities as well as proper f-wave latency. Nevertheless, myopathic changes in EMG of the quadriceps femoris muscle were found: spontaneous activity, in parts small nonpolyphasic motor unit action potentials (MUAPs, lowest 0.3 mV), early dense interference pattern. The muscle ultrasound demonstrated hyperechogenicity in the proximal muscles, especially the quadriceps femoris muscle (modified Heckmatt Scale [5]: 2/4 both sides; see Figure 1(d)). Overall, this is indicative of a myopathy. Interestingly, an MRI of the upper leg muscles showed normal T1, T2-TRIM, and Dixon with contrast agent sequences (see Figures 1(a), 1(b), 1(c)). The absence of signs of myotonia in the examination, as well as the EMG showing no myotonic discharges, did not suggest proximal myotonic myopathy. Endocrine causes (e.g., hypothyroidism and hyperparathyroidism), medication, and addictive substances were unlikely. At the time of respiratory insufficiency, the patient had not taken any medications. The history and drug screening were not indicative of substance abuse. Furthermore, laboratory results obtained during the ICU stay showed normal thyroid levels and no indication of a calcium metabolism disorder. Due to the described acute course, a muscle biopsy of the right vastus lateralis muscle was performed in regard of other differential diagnosis like myositis. Finding revealed nonspecific neurogenic variations in muscle fiber caliber and marginal, crescent-shaped, pale blue-gray homogeneous deposits on toluidine blue staining (see Figure 2). These deposits, ultrastructurally consistent with beta-glycogen particle accumulations, suggested a glycogen storage disease. However, a prior measurement of alpha-1,4-glucosidase enzyme activity was normal, and the acid phosphatase stain in the muscle biopsy was negative. Both findings argued against Pompe disease, leading an expansion of the genetic testing for a glycogen storage. Finally, genome sequencing revealed a missense variant (c.230G > A, p.Arg77Gln) and a frameshift variant (c.284del, p.Cys95Phefs∗241) in *MEGF10* (ENST00000503335.7), present in a compound heterozygous state indicative for an autosomal-recessive MEGF10 myopathy. The targeted follow-up revealed that—despite largely normal motor milestones—the patient slightly lagged behind peers and experienced recurrent morning headaches since early childhood, which in the overall context suggest a long-standing nocturnal hypoventilation. Overall, respiratory insufficiency, swallowing difficulties, proximal symmetrical paresis, and scoliosis are associated with MEGF10 myopathy, and both the clinical and genetic results led to the diagnosis.

For the following 2 years, the patient has been experiencing a stable disease course, receiving noninvasive ventilation at night (mode: pressure support ventilation, IPAP 20 mbar, EPAP 6 mbar) and continuous oxygen monitoring (no long-term oxygen therapy during day), leading to headache relief, with stable muscle strength and well-managed muscle pain with lamotrigine.

## 3. Discussion

Our case report demonstrates that in cases of seemingly unclear symptoms initially presenting with sudden respiratory insufficiency, a thorough evaluation—including consideration of rare muscle diseases beside of several internal causes—is worthwhile.

In general, respiratory failure can be a major syndrome in neuromuscular diseases and can be the initial presentation (e.g., about 14% in myasthenia gravis [6] and 3% in ALS [7]). In some muscle diseases, it is even part of the clinical description of typical symptoms (e.g., nemaline rod myopathy: muscle weakness, respiratory insufficiency, and dysphagia [8]). Typical daytime symptoms are hypersomnolence, dyspnea (positional, exertional, or otherwise unexplained), tachypnea, slow speech/dysarthria, weak cough, and fatigue [1]. Nocturnal symptoms including orthopnea, sleep disruption, and headache upon awakening are quite common [1]. Interestingly, in the target follow-up, our patient described unexplained regularly occurring headache especially in the morning, what remained unexplained for a long time. Moreover, the headache relieved by receiving noninvasive ventilation at night. This aspect illustrates that, in retrospect, a long-standing symptomatology likely existed for many years, and that the apparently sudden onset of respiratory insufficiency merely represented the visible manifestation of an underlying, progressive condition, pointing to a possible hereditary muscle disease like MEGF10 myopathy.

MEGF10 myopathy is an ultrarare autosomal-recessive myopathy, often presented with dyspnea (up to respiratory failure), dysphagia, muscle weakness, and hyporeflexia/areflexia as well as with cleft palate and scoliosis [2–4]. The age of onset varies, ranging from childhood to adulthood [2]. Muscle biopsy can show unspecific muscle damage (fiber size variation, internal nuclei, and fibrosis) or myofibrillar alterations (whorled fibers, cytoplasmic inclusion, and minicores) [9]. However, in addition to unspecific muscle damage, our muscle biopsy showed glycogen mimicking deposits, which, to the best of our knowledge, have not been previously described in the context of MEGF10 myopathy. This seems to be an unrelated finding, as genetic testing did not indicate any signs of a glycogen storage disorder. Therefore, we consider this result as an epiphenomenon related to the disease rather than a direct manifestation of a glycogen metabolism disorder. The MEGF10 gene itself is located on chromosome 5q23.2 and encodes a transmembrane protein involved in the Notch signaling pathway [4]. It is highly upregulated during myoblast proliferation, playing a crucial role in the development and regeneration of skeletal muscle [4, 10]. In the context of MEGF10 myopathy, the severity of the symptoms is dependent on the genetic findings [2, 9]. The missense mutation (c.230G > A, p.Arg77Gln) in the patient has previously been associated with a milder phenotype (e.g., [11]). Furthermore, a frameshift variant (c.284del, p.Cys95Phefs∗241) was detected, which predicts a premature stop codon and disruption of the reading frame, suggestive for an earlier clinical manifestation. Other frameshift variants in MEGF10 myopathy are associated with an early onset (e.g., [12]). In this case, the combination of the two mutations is likely to result in a partial function of the protein. It could explain why severe symptoms may have become apparent relatively late in early adulthood.

In conclusion, rare muscular syndromes should be considered in cases of initial respiratory insufficiency, particularly when additional neurological deficits such as muscle pain emerge during the clinical course. Accurate delineation of the myopathy spectrum is essential, as demonstrated in the present case. Findings such as those in the muscle biopsy or the combination of different mutations—which may individually be associated with varying clinical courses—may reveal patterns that have not been described before.

## Data Availability

The data that support the findings are available from the corresponding author upon reasonable request.

## Nomenclature

TLC: Total lung capacity
FVC: Forced vital capacity
RV: Residual volume
P0.1: Occlusion pressure 0.1 s
MIP: Maximal inspiratory pressure
GBS: Guillain–Barré syndrome
CIP: Critical illness polyneuropathy
ICU: Intensive care unit
MND: Motor neuron disease
EMG: Electromyography
MUAPs: Motor unit action potentials
